# Saturation flow rate analysis for special width approach lanes: An empirical study in Karlsruhe, Germany

**DOI:** 10.1371/journal.pone.0272503

**Published:** 2022-08-23

**Authors:** Jingqi Xu, Kevin K. Kigen, Dalin Xu, Shilin Wang, Min Gu, Xinyu Liu, Jing Zhao

**Affiliations:** 1 Department of Transportation Engineering, University of Shanghai for Science and Technology, Shanghai, China; 2 People’s Government of Gaohang Town, Shanghai, China; 3 S S Mehta & Sons Limited, Nairobi, Kenya; 4 Jiangsu Automation Research Institute, Lianyungang, Jiangsu Province, China; 5 Shanghai Municipal Engineering Design Institute (Group) Company Limited, Shanghai, China; Rutgers University, UNITED STATES

## Abstract

The special width approach lane (SWAL) is a newly proposed unconventional design, whereby a wide approach lane is divided into two narrower lanes. The design entails the use of a single lane by two passenger cars or one heavy vehicle. Such design has been applicated at signalized intersections of Karlsruhe, Germany. This paper focuses on the saturation flow rate analysis since most existing studies on such design rely on the default highway capacity manual (HCM) values. Saturation flow rate data was collected at four SWAL design based signalized intersections with procedural steps of the HCM 2010 using the video camera. The two-sample t-test was performed to explore the potential influencing factors, and then the non-linear regression analysis was conducted to estimate the saturation flow rate of SWAL. The proposed model can effectively depict the saturation flow rate with lane marking, presence of cyclists, and rainfall being the influencing factors. The overall accuracy of the proposed model is about 95%. The results indicate that the three influencing factors are independent of each other. The existence of cyclists and rainfall lead to a decrease in the saturation flow rate, while the lane markings can improve the saturation flow rate. Moreover, the SWAL works well in Karlsruhe, Germany. The model predicts a base saturation flow rate value of 1652 pcu/h/ln, which is plausible with comparison of the base saturation flow rate recommended in the German Highway Capacity Manual.

## 1. Introduction

Traffic menace is a ubiquitous and inescapable condition in large metropolitan cities. This is a universal problem even for well-planned cities, which seems to be a major obstacle to smooth sailing traffic. Presence of far too many cars in the roads predominantly due to inadequate infrastructural transit systems leads to build up of congestions. This is generalized as there being an imbalance in demand and supply in the traffic system.

Transportation departments together with policy makers in urban planning and management came up with traditional ways of solving traffic predicaments with emphasis on capacity improvement such as; expanding already existing infrastructure by building new structures. With time however the space has shrunk in major cities and expansion is no longer a viable and an attractive option as it used to be. Engineers and researchers have established Transportation Management Systems options in coming up with modern means of improving the capacity. This has led to emergent of numerous unconventional intersection designs such as exit lane for left turns, displaced left turns, tandem intersections, median U turn, Jug Handle, Superstreets and use of pre-signals [1–4].

Our concentration and drive for this study is the unconventional design known as Special Width Approach Lane (SWAL) by Zhao et al. [5]. This a tentative design whereby a wide approach lane is divided into two narrower lanes and the lanes are independent of each other in their operation. The design entails the use of a single lane by two passenger cars or one heavy vehicle such as a bus. This depends on the composition of the traffic. With the reduced space in cities, expansion of lanes is an issue when it comes to increasing the level of service of an intersection. SWAL helps increase the number of approach lanes while retaining the operational safety especially for the heavy vehicles. This leads to an increase in the capacity and an improvement in saturation flow rate of the approach lane.

Various aspect of the width design of traffic lanes has been studied. Chang et al. [6] analysed the relationship between the lane width and saturation flow rate, lateral safety distance and traffic crash rate. The results show that optimal lane width for four-legged intersections is 3.1m and for a limited road resources a lane width of 2.8 m is proposed. Parkin and Meyers [7] found out that the presence of cyclist lane at the edge of the approach lane may lead to drivers tending to reduced speeds while passing close to the cyclist lane. Anvari et al. [8] proposed a three layered microscopic mathematical model to represent behaviour of pedestrians and vehicles in shared space layouts and on a simulated traffic scenario with success in improving the safety and raising the quality of urban arterial designs. More specifically, for the SWAL design, the operation of vehicle was explored based on a microscopic traffic flow model with emphasis on the driving behaviour and consideration of lane selection, heavy vehicles volume and length of the SWAL as influencing factors.

Much has been done by specialists in the field, however, the saturation flow rate for SWAL geometric design layout has not yet been carefully studied. The ideal saturation flow rate is assumed in the existing studies.

Saturation flow rate is an essential parameter of the geometric design, signal control, and level of service evaluation at signalized intersections [9–11]. Researchers have carried out studies on various adjustment factors present in the intersections and their influence on the saturation flow rate [12–14]. The common influencing factors, such as area type, lane width, heavy vehicles, grade, turnings, pedestrians and bicycles, have been considered in the manuals, such as the highway capacity manual (HCM) [15]. Other concerned adjustment factors for saturation flow rate include the weak-lane-disciplined mixed traffic stream [16–19], short lanes [20], left-turn waiting area [21], adverse weather conditions [22], lighting conditions [23], access traffic [24], guide line markings [25], signal countdown devices [26], and autonomous driving environment [27]. Recently, with the wider use of the unconventional intersection design, the saturation flow rate of the lanes under the unconventional design were also explored based on empirical data. For the tandem intersections, the saturation flow rate adjustment model was developed with influencing factors being unequal distribution of traffic, red light violations at the pre-signal and incomplete discharge of vehicles at the sorting area [28]. For exit lanes for left-turn intersections, the saturation flow rate was adjusted with the consideration of five influencing factors, including the median opening blockage, demand starvation, multilane interference, conflict with opposing vehicles, and lane changing [29]. For continuous flow intersections, study shows that a reduction in the saturation flow rate for the left-turn, through movement, and left-turn at the pre-signal with consideration of predictor variables being heavy vehicles, proportion of lane changing vehicles, and length of the displaced left-turn lanes [30].

Although numerous research has been conducted on saturation flow rate and the influencing factors for the various geometrical configuration, there is absence of research on the saturation flow rate for signalized intersections with the special width approach lane phenomenon. Previous study has shown the promising effectiveness in improving operational efficiency as a whole. However, we also know that the saturation flow rate cannot be the same as on normal traffic lanes. To fully understand the operational efficiency of the SWAL, a proper saturation flow rate analysis with field data is proposed. This study aims to carry out the saturation flow rate analysis of SWAL using collected field data in the South Western city of Germany known as Karlsruhe.

This research is paramount in understanding the functioning and workings of signalized intersections with special width approach lane (SWAL) design for the approach lanes, both in terms of saturation flow rate and capacity utilization of the intersection. A deep detailed discussion is lucking for the operational performance of the SWAL. The findings of this study will redound to the benefit planning and design of the unconventional signalized intersection with shrinkage in the approach lanes width. Research on the operational analysis of the signalized intersections inculcates an inductive thinking which will lead to proper conclusions being drawn on the best optimal design possible for the SWAL with consideration of level of service, saturation flowrate, travel speed and operational safety of the facility. Moreover, the findings will help in knowing what influencing factors affects the driver’s behavior and their SWAL understanding and usage, effects of pedestrians and cyclists on vehicles on reduced lanes and willingness of drivers to drive side by side under different conditions. Thus, proper conclusions can be drawn on the what best can be done to optimize the usage of special width lanes with improved level of service and reduced delay times.

The paper is organized into the following sections: section 2 details the materials, data collection and the analysis methodology. Section 3 presents the results of the findings. Section 4 discusses the shortcomings and solutions in the implementation of SWAL design. Section 5 summarizes by concluding and proposing future works.

## 2. Data collection and analysis method

To determine the saturation flow rate of a signalized intersection with SWAL, careful consideration is taken in selection of the location for data collection, which is a fundamental step in this study.

### 2.1 SWAL design introduction

**Fig 1:**
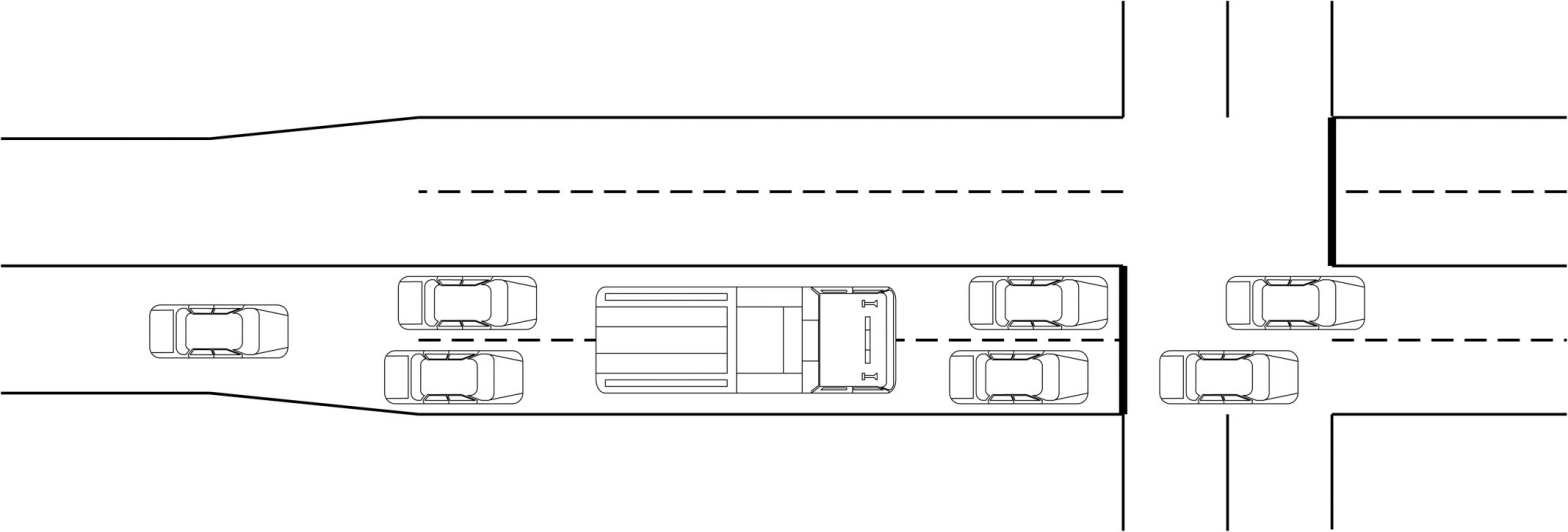
Layout design of SWAL.

Since the SWAL design is newly proposed, we introduce such design briefly in this section. Space has become the upcoming issue in major signalized intersections. With modern development fast out pacing the existing facilities, engineers have been tasked with coming up with unconventional designs such as special width approach lane (SWAL). SWAL is a geometrical modification of an intersection where by, two narrow approach lanes are dynamically utilized by two passenger car units or one heavy vehicle such as truck or a bus, as shown in Fig 1. The width of SWAL is 5.2 m. The width of each sub-lane on SWAL guided by the AASHTO standards is a minimum of 2.6 m for small vehicles. The heavy vehicles will use both the two lanes concurrently since its width is much larger compared with the small vehicles.

If the approaching vehicles are all passenger vehicles the phenomena serve to act as two independent lanes. If the normalcy of traffic behavior is to be expected, heavy vehicles will integrate with the passenger cars in several traffic situations. If the number of the heavy vehicles is on the higher side this will result in inadequate usage of the SWAL. Different movements in the SWAL have impacts on its operational efficiency. This is guided by how well the lane assignments match the arrival patterns and traffic composition.

#### 2.1.1 Data collection

**Fig 2:**
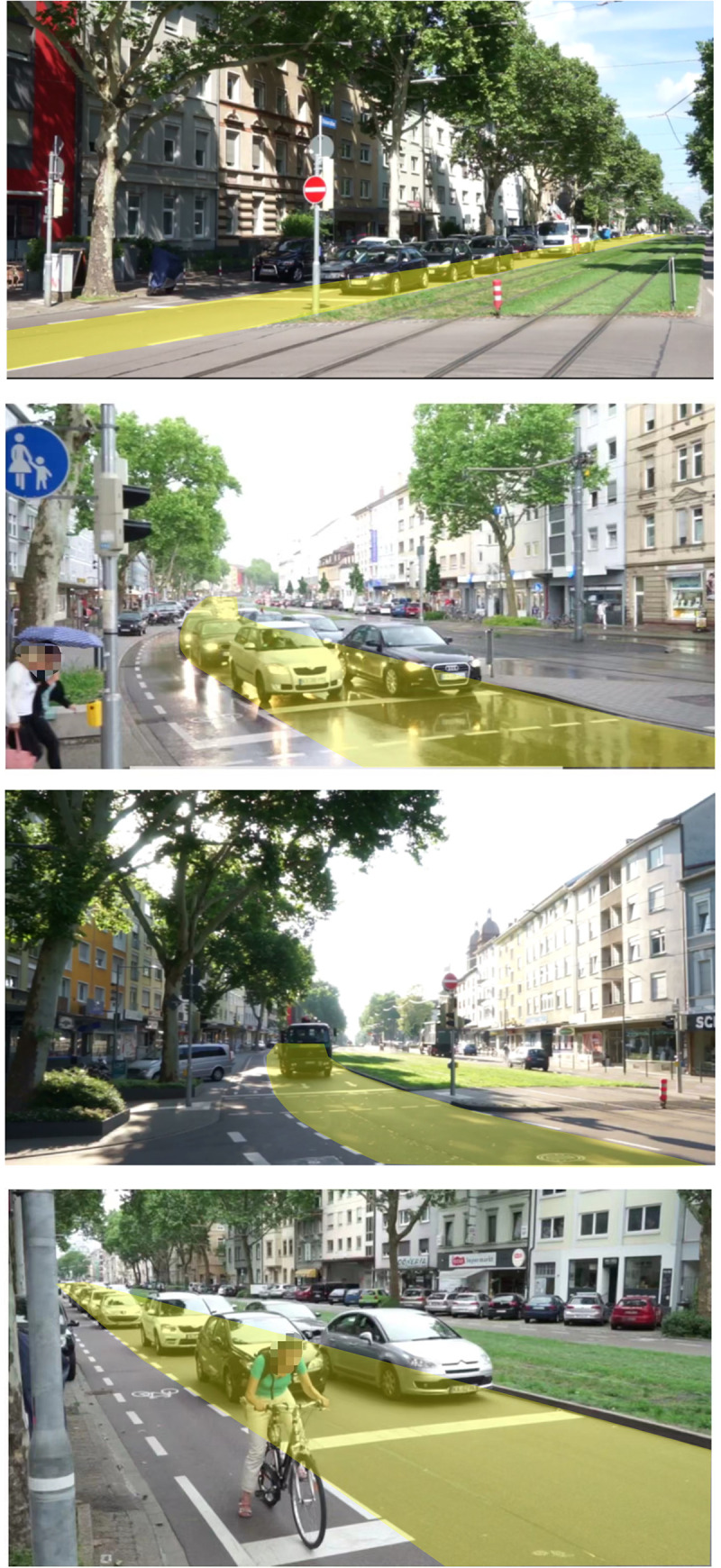
Data collection of the SWAL approaches in Karlsruhe, Germany. (a) Karlstrasse- Amalienstrasse. (b) Rheinstrasse–Philippstrasse. (c) Rheinstrasse–Nuiststasse. (d) Rheinstrasse—Am Entenfang.

To the authors knowledge, Karlsruhe a mid-level urban city at the south western part of Germany is the only city with the presence of SWAL design signalized intersections. To eliminate the interference of other factors, a thorough reconnaissance of the intersections was considered with the gradient sufficiently being flat, no bus stops, and the queues being long enough to facilitate the collection of sufficient amounts of data. The intersections of Karlstrasse- Amalienstrasse, Rheinstrasse–Philippstrasse, Rheinstrasse–Nuiststasse, and Rheinstrasse—Am Entenfang were identified as suitable for our data collection depicted in Fig 2. The SWAL has been highlighted in yellow. All the SWALs in the surveyed sites are exclusive through lanes.

A modern video detection device (video camera) was mounted with a heavy polythene cover to prevent it from the harsh climatic conditions such as heavy rainfall. The video camera was setup on the right side of the approach lanes of the intersection, which is a few meters from an observable point of view of the stop line. This presented a good view of the entire approach lane from the stop line, with close to 200 meters of observable approach lane sight. The video recordings were carried out in the morning peak hours of 8am to 10am and evening peak hours 4pm to 6pm for three working days.

The recorded videos were used in a data processing tool software [31–33] to extract the headways and thus calculation of the saturation flow rates. The time is recorded when the front axle of the lead vehicle touches the stop line and it is stopped when the following vehicles front axle touches the same position in the stop line. The difference in time is recorded as the headway. This is done subsequently for all the vehicles in the queue. Data from traffic scenarios such as vehicles stopping midway or halfway through queues or at stop line, motorists forming part of the queue in the approach lane, pedestrians interfering with vehicles was discarded.

For these signalized intersections, the approach lane of each leg is divided into two lanes; lane 1 and lane 2 where by lane 1 is the outer lane bordering the cyclist’s lane and while lane 2 is the inner lane. Some intersection locations are clearly lane marked while others are not marked. Other considered potential influencing factors include the existence of cyclists and rainfall. The influencing factors are simultaneously recorded. A sample of 1743 vehicles was collected for the four signalized intersections and was deemed sufficient for the analysis.

#### 2.1.2 Saturation flow rate calculation

Saturation flow rate is a measure that is used extensively in traffic flow analysis to determine, evaluate the performance of an intersection and how to optimize traffic flow. The geometrical and signal design of signalized intersections relies heavily on the results of the saturation flow rate. Saturation flow rate as defined by the Highway Capacity Manual is the equivalent hourly rate at which vehicles can pass through an intersection approach under prevailing conditions in vehicles per hour of green assuming the green signal is always available and no lost times are experienced.

Headway is measured in seconds. This is the distance in seconds between successive predetermined points of vehicles in a queue on a lane at a point of interest. The measurement of the headway was carried out with arbitrary common points of the vehicles such as; the front bumper of the lead vehicle to the front bumper of the following vehicle. The headway will tend to be different at different points of the queues. A shorter headway indicates a longer gap or distance between the successive vehicles. At the start of the queue, it will be longer than the preceding headways, but will normalize after about the fourth to sixth vehicles [15]. Therefore, the headways of the queuing vehicles after the fourth vehicle are used, which is referred to as saturation headway. The saturation flow rate is thus computed as the number of seconds per hour divided by the constant saturation headway as shown in the Eq (1) [15]. The process is repeated on all the queues of each cycle and in all the intersections.

$$s=\frac{3600}{h_{s}}$$ (1)

where *s* is the saturation flow rate of SWAL, veh/h/ln; *h*_*s*_ is the surveyed average saturation headway in a cycle, s.

#### 2.1.3 Analysis method

The data analysis procedure includes the following two steps. The first step is to check whether the effects of the potential influencing factors on the saturation flow rate of the traffic lanes at SWAL approaches are significant. Then, the effects are quantified based on non-linear regression analysis.

*2*.*1*.*3*.*1 Significance analysis*. The two-sample t-test is used for purposes of determining the statistically significant difference of the potential influencing factors, including the existence of cyclists, the lane markings, and the rainfall. For the two-sample t-test, the data should be in the form of one independent categorical variable that has two groups and one continuous dependent variable. The major assumptions that are considered are: the data should be continuous or follows an ordinal scale, the data are sampled randomly and is normally distributed. The dependent variable which is the saturation flow rate should also be approximately distributed within each group. The test is a robust test in accordance with the assumption of normality. This means that some deviation away from normality has significantly less influence on the error rate.

There are two ways of determining statistical significance using the independent T test. First, by checking the probability value (*p* value or sig 2 tailed). If *p* < 0.05, it is statistically significantly different. It can also be done using the confidence level. If it crosses zero that implies it passed from negative to positive (- to +), the data are not statistically significantly different.

*2*.*1*.*3*.*2 Regression analysis*. Normally, the relationship between the saturation flow rate and the predictor factors is not linear. The regression to be performed on the dependent variable is modelled as a non-linear function of the model parameter of the independent variables. The analysis will help us understand by what extent the predictor variables will significantly affect the saturation flow rate either positively or negatively.

The first step is to come up with a model expression which is based upon the tried and tested methodology by other researchers in calculation of the saturation flow rate. The value of the coefficient can be correctly interpreted only if the correct model has been fitted, therefore model establishment is of great importance.

The proposed model is based on the HCM saturation flow rate model [15]. From the framework in HCM 2010 [15], the saturation flow rate is calculated by multiplying the base saturation flow rate by the specified geometric, signal, and traffic adjustment factors that affect the lane. It is assumed that the adjustment factors are independent with each other. Following the saturation flow rate calculation framework of HCM 2010 [15], Eq (2) is used to determine the saturation flow rate of SWAL with the effects of the influencing factor.

$$s=s_{0}f_{c}f_{l}f_{r}$$ (2)

where *s*_0_ is the base saturation flow rate for SWAL, pc/h/ln; *f*_*c*_, *f*_*l*_, and *f*_*r*_ are the adjustment factors of the existence of cyclists, the lane markings, and the rainfall, respectively.

For the effect of cyclists, we assume that the presence of the cyclists may have a negative effect on the saturation flow rate. It means the adjustment factor is lower than 1 when the cyclists are present, while it should be equal to 1 when the cyclists are absent. Therefore, the adjustment factors of the existence of cyclists can be formulated as Eq (3).

$$f_{c}=1+(\alpha_{c}-1)P_{c}$$ (3)

where *α*_*c*_ is the parameter to be regressed, which reflects the negative effect caused by the presence of the cyclists; *P*_*c*_ is percentage of the presence of the cyclists. Please note, the value of *P*_*c*_ for the collected data is either 1 (the presence of the cyclists) or 0 (the absence of the cyclists).

For the lane marking, it is a binary parameter. The existence of the lane marking means that the two narrow lanes on the SWAL are clearly divided. We assume that the presence of the lane marking may have a positive effect on the saturation flow rate. It means the adjustment factor is lower than 1 when the lane marking is absent, while it should be equal to 1 when the cyclists are present. Therefore, the adjustment factors of the lane marking can be formulated as Eq (4).

$$f_{l}=1+(\alpha_{l}-1)(1-M_{l})$$ (4)

where *α*_*l*_ is the parameter to be regressed, which reflects the negative effect caused by the absence of the lane marking; *M*_*l*_ is the presence of the lane marking, 1-yes, 0-no.

For the effect of the rainfall, we assume that the presence of the rainfall may have a negative effect on the saturation flow rate. It means the adjustment factor is lower than 1 when the rainfall is present, while it should be equal to 1 when the rainfall is absent. Therefore, the adjustment factors of the rainfall can be formulated as Eq (5).

$$f_{r}=1+(\alpha_{r}-1)R$$ (5)

where *α*_*r*_ is the parameter to be regressed, which reflects the negative effect caused by the rainfall; *R* is percentage of the presence of the cyclists, 1-yes, 0-no.

In summary, the general equation to depict the saturation flow rate is shown in Eq (6). The parameters to be fitted include $s_{0}^{\prime }$, *α_c_*, *α_l_*, and *α_r_*. Please note that the multiplicative model is used, which assumes that the three influencing factors are independent with each other. This assumption will be validated after the regression analysis of the model.


$$s=s_{0}[1+(\alpha_{c}-1)P_{c}][1+(\alpha_{l}-1)(1-M_{l})][1+(\alpha_{r}-1)R]$$ (6)

## 3. Saturation flow rate analysis results

The results of the saturation flow rate analysis and the discussion of the plausibility of the results are presented in this section.

## 3.1 Significance analysis

The statistical results of the significance analysis are shown in Table 1. There is a statistically significance mean difference on the cyclists as an influencing factor whilst for the lane marking and rainfall there is not much statistically significance difference.

**Table 1:** Statistical mean of the saturation headway.

Factor	Category	Number of samples	Mean	Std. Deviation	Std. Error Mean
Cyclists	1	132	3.362	1.619	0.141
	0	1610	2.709	1.587	0.040
Lane marking	1	377	2.645	1.352	0.070
	0	1365	2.790	1.659	0.045
Rainfall	1	219	2.780	1.467	0.099
	0	1523	2.756	1.617	0.041

Table 2 depicts the results of Levene’s Equality of Variance test of homogeneity. For the cyclists since the probability value is less than 0.05, it implies that there is unequal variances and violation of the assumption of homogeneity of variance. The values of the second row will be used. The *p* value is still less than 0.05 meaning the mean values of the cyclists is statistically significantly different thus accept the null hypothesis that there is indeed statistically significant difference in the cyclist’s group.

**Table 2:** Significance test.

Factors		Levene’s Test for Equality of Variances		t-test for Equality of Means						
		F	Sig.	t	df	Sig.	Mean Difference	Std. Error Difference	95% Confidence Interval of the Difference	
									Lower	Upper
Cyclists	Equal variances assumed	1.415	0.234	4.540	1740	0.000	0.653	0.144	0.371	0.936
	Equal variances not assumed			4.462	152	0.000	0.653	0.146	0.364	0.943
Lane marking	Equal variances assumed	2.507	0.114	-1.565	1740	0.118	-0.145	0.093	-0.328	0.037
	Equal variances not assumed			-1.756	720	0.079	-0.145	0.083	-0.308	0.017
Rainfall	Equal variances assumed	.007	0.934	.212	1740	0.832	0.025	0.116	-0.202	0.251
	Equal variances not assumed			.228	299	0.820	0.025	0.107	-0.187	0.236

The *p* value of lane marking is 0.320 and that of rainfall is 0.643, which are both greater than 0.05 making it to be statistically not significantly different. Thus, the null hypothesis is rejected and an alternative is that the data are not statistically significantly different.

Moreover, the confidence interval for the cyclists as shown in table ranges from 0.090 to 0.139 thus does not cross zero while for lane marking and rainfall the confidence interval both cross zero. This further confirms that the cyclists are statistically significantly different while the lane marking and rainfall is not statistically significantly different.

### 3.2 Regression analysis

After coming up with reasonable starting points, the model ran iterations and stopped after 10 runs and a convergence was established for the set parameters, as shown in Table 3.

**Table 3:** Iteration procedure results.

Iteration number	Residual sum of squares	Parameter			
		$s_{0}^{'}$	*α*_*c*_	*α*_*l*_	*α*_*r*_
1.0	1387295707.393	1500.000	0.500	0.500	0.500
1.1	401058846.271	1640.761	1.208	1.044	1.535
2.0	401058846.271	1640.761	1.208	1.044	1.535
2.1	101098071.038	1647.425	0.970	0.988	1.006
3.0	101098071.038	1647.425	0.970	0.988	1.006
3.1	100080844.477	1652.477	0.943	0.986	0.970
4.0	100080844.477	1652.477	0.943	0.986	0.970
4.1	100080833.616	1652.570	0.943	0.986	0.970
5.0	100080833.616	1652.570	0.943	0.986	0.970
5.1	100080833.616	1652.570	0.943	0.986	0.970

Table 4 displays the overall parameter estimation values. The confidence limits depict the lower bound and upper bound values of the proposed model parameters. Table 5 shows the correlation of influencing factors amongst themselves and whether they have significant influence on the dependent variable which is the saturation flow rate. The higher the correlation value which is maximum at 1 depicts a robust relationship of influencing factors amongst each other while a low correlation value of closeness to 0 means the correlation is highly unlikely among the influencing factors. A correlation value of lower than 0.3 (range of 0.0 to 0.3) in value means the influencing factors are highly unrelated in influencing the outcome of the dependent variable [34] thus deemed to be negligible. In Table 6, we find all the correlation values between two influencing factors are lower than 0.3. Therefore, the assumption of the proposed model (Eq (6)) that the influencing factors are independent with each other is true. The use of the multiplicative model is reasonable.

**Table 4:** Parameter estimation.

Parameter	Estimate	Std. Error	95% Confidence Interval	
			Lower Bound	Upper Bound
$s_{0}^{'}$	1652.570	12.515	1628.023	1677.116
*α*_*c*_	0.943	0.013	0.917	0.969
*α*_*l*_	0.986	0.009	0.969	1.003
*α*_*r*_	0.970	0.011	0.948	0.991

**Table 5:** Correlations of influencing factors.

	*f*_*c*_	*f*_*l*_	*f*_*r*_
*f*_*c*_	1.000	-0.004	0.028
*f*_*l*_	-0.004	1.000	-0.196
*f*_*r*_	0.028	-0.196	1.000

**Table 6:** Calculated Saturation flow rate.

Intersections	Lanes	Bicycles (*P*_*c*_)	Lane marking (*M*_*l*_)	Rainfall (R)	Adjust values	Saturation flow rate (veh/h)		
						Collected	Calculated	Relative error
Karlstrasse–Amalienstrasse	Lane 1	1	0	0	0.930	1588	1537	3.21%
	Lane 2	0	0	0	0.986	1522	1629	7.03%
Rheinstrasse–Philippstrasse	Lane 1	1	0	1	0.902	1421	1490	4.86%
	Lane 2	0	0	1	0.956	1465	1581	7.92%
Rheinstrasse–Nuiststasse	Lane 1	1	1	0	0.943	1565	1558	0.45%
	Lane 2	0	1	0	1	1736	1653	4.78%
Rheinstrasse–Am Entenfang	Lane 1	1	0	0	0.930	1642	1537	6.39%
	Lane 2	0	0	0	0.986	1747	1629	6.75%

### 3.3 Plausibility analysis

The saturation flow rate can be estimated using Eq (6) with the parameters calibrated in Table 4. The calculation results are shown in Table 6. The comparison of the collected saturation flow rate and the proposed model shows that there is the difference between the two is lower than 8% in all surveyed traffic lanes. The average relative error is 5.17%. Moreover, the paired samples significance test (Table 7) shows that there is no significant difference collected saturation flow rate and the calculated one (*p* value = 0.797 > 0.05). It indicates that the model is in line with the field data results.

**Table 7:** Paired samples significance test.

Paired differences					t	df	Sig. (2-tailed)
Mean	Std. deviation	Std. error mean	95% Confidence interval of the difference				
			Lower	Upper			
9.00000	95.249	33.675	-70.630	88.630	0.267	7	0.797

Another interesting finding is about the base saturation flow rate. The proposed model recommends a value of 1652 pcu/h/ln for the base saturation flow rate of the SWAL. It makes sense that the base saturation flow rate of the SWAL is slightly lower than that of normal traffic lanes recommended in the capacity manuals. E.g., for a medium size city such as Karlsruhe with a mean population of 250,000 to 300,000 people a saturation flow rate of 1750 pcu/h/ln to 1950pcu/h/ln is the default value recommend by the HCM 2010 [15]. The German Highway Capacity Manual [35] recommends a base saturation flow rate of 2000 pcu/h/ln and an assumed base saturation headway of 1.8 seconds used for the base condition. It means that the adjustment for the SWAL can be calculated by dividing this saturation flow rate the German saturation flow rate to get an adjustment factor of 0.826.

## 4. Discussion

### 4.1 Shortcomings in implementation of SWAL design

Potential problems and shortcomings of the SWAL can be divided and discussed in three major groupings. These are problems arising from the running efficiency of the design, the safety of SWAL and the traffic laws and discipline. On matters of efficiency, it will be further subdivided to discuss; the driving behavior, the type of vehicles, turning movements in the intersection and the signal timing. The safety of the SWAL will dwell on the vehicles, pedestrians, cyclists and non-motorists, while on traffic laws and discipline will talk about speed limits, poor observation of traffic signs.

#### 4.1.1. Visibility of the SWAL intersection

The inability to view the entire SWAL design intersection is possible when approaching the intersection from a major or minor road. This can be a very serious problem for the drivers, motorists and cyclists. The intersection should be visible enough to allow for all the traffic road users enough time to reach and adjust themselves accordingly especially when on the entering, transition and SWAL segments whereby lane selection and lane changing take place. Different segments of the SWAL design might not be visible especially if the lane marking is not adequately carried out. The provision of ample time to adjust can be done by provision of enough stopping sight distance under the guidance of each countries respective design guidance and design manuals.

#### 4.1.2. Collisions with motorists and non-motorists

Road users such as pedestrians and cyclists are highly vulnerable to serious injuries at SWAL design intersections. Most of the accidents that happen in intersections are due to drivers. Right turns and left turns are the major culprits. Because these type of turns leads to side impact collisions that are severe when compared to head to head collisions. Side impact occurs at the sides of vehicle where there is no cushion or protective covers. Situations where drivers focus on the lead vehicles and pay little attention on cyclists’ lane or pedestrians waiting to cross may lead to accidents. High speeds in approaches and through ways which leads to increase in likelihood of traffic incidents.

#### 4.1.3. Lack of guidance for road users

SWAL design with no lane marking as seen from chapter 3 as an influencing factor of the saturation flow rate analysis leads to a poor operational ability and low saturation flow rate especially for new drivers. SWAL design with no lane markings or insufficient markings such as barricades, channelization markings, delineators for purposes of navigation of the intersection is a critical issue that should be taken into uttermost consideration. Lack of lane marking can lead to motorist’s confusion especially for the new drivers to the SWAL design or new drivers of vehicles. The pedestrians and cyclists too can get overwhelmed with lack of pavement lane marking especially for cyclist’s lane and pedestrian’s crossing. This can get worse during extreme weather conditions such as heavy rainfall, hail or heavy snow cover.

#### 4.1.4. Driving behaviour

Driving behavior will ultimately change throughout the entire SWAL intersection. The entering segment will experience the normal car following behavior since the width is still the same as that of the conventional intersection. In the transition section where the lane is divided into two narrow lanes the drivers need to choose one of the two lanes. The lane selection and lane change leads to some slight confusion amongst the drivers on which car to follow. On the special width lane segment the reduced lanes can bring about lateral scratches to vehicles especially for drivers who are inexperience or new to the design. The exiting section has no such problems as vehicles will be served on the first come basis thus most of the conflicts are avoidable. New drivers can experience some slight challenges adjusting to the SWAL and this can lead to serious delays and thus hinder the operational ability of the intersection.

#### 4.1.5. Illegal maneuvers

Most drivers are tempted to perform illegal maneuvers while using the intersection. SWAL designed intersection unfortunately is not designed for such illegal movements because of the reduced width. Sudden lane changing in the special width lane segment can lead to collisions, lateral scratches and far more damage to those involved. Inexperienced drivers can make illegal U turns and can lead to head on collisions of vehicles.

### 4.2 Solutions to problems in implementation of SWAL

Due to complex environment for the implementation of the SWAL in real-world, the following considerations might bring benefit to the operation efficiency.

#### 4.2.1 Visibility of SWAL

To solve the problem of the visibility of the SWAL adequate stopping sight distance should be provided. The American Association of State Highway and Transportation Officials (AASHTO) [36] provides guidance on the stopping sight distance and should be implemented in the design of the SWAL as well. A proper layout of the traffic road signs should be installed and erected further upstream such as the stop sign. This can be done to aid the view of motorists in situations where the design has been implemented on a signalized intersection. General conflicts will be avoided between the vehicles at intersections even though the total avoidance of conflicts depends on the capabilities and response of the drivers. The sight distance that can be considered safe should be related to the velocity of the vehicles and the perception reaction time until it breaks. All vehicles approaching the SWAL should have unblocked and clear sight of view among each other to prevent collisions. This will make the drivers be able to see each other well and be able to slow down at the intersection.

#### 4.2.2 Collisions with motorists and non-motorists

Pedestrians and Bicyclists are ever present in every intersection. Conflicts and collisions are bound to happen at the course of usage of SWAL design. Pedestrians and cyclists travel where they possibly can and use the facilities of intersection regardless of the design and safety standards consideration. The best solution of the pedestrians and cyclists is to have them in mind when designing and implementing the SWAL intersection. A thorough reconnaissance of the subject intersection is crucial to observation of how the cyclists, pedestrians and motorists use it. A study should be conducted and survey carried out on the views of if the pedestrians and motorists would use the proposed design rather than alter their movements in the intersection.

#### 4.2.3 Road guidance for road users

Proper and comprehensive lane marking should be carried out for the entire intersection. Provision of dotted markings for the separation of the reduced width lane together with directional signs should be enough for the guiding of drivers and other motorists in the SWAL segment. This will inadeptly lead to reduce conflicts and side by side harmoniously driving. A proper bicycle lane should be marked with different colour to distinguish it from the normal corridor lanes and the pedestrians path bounds should be highlighted so as not to interfere with the bicycle lane. Luminescent paint should be used for the purposes of night visibility especially for the transitional segment and SWAL sections.

#### 4.2.4 Driving behaviour

The driving behaviour is affected by many factors such as duration and time of the day, surrounding traffic environment, weather and occasions and the site where the SWAL design is located. The drivers should be properly guided by signs that indicate different segments of the SWAL for ease in transition from one area to the next. Even though the drivers cannot drive efficiently on narrower lanes, it is possible and with benefits of increased saturation flow rate and capacity.

#### 4.2.5 Illegal manoeuvres

To avoid illegal manoeuvres, education on the use of the SWAL is crucial for the effectiveness and full utilization of the design intersection. Since it is and will be a novel design for most regions of the urban cities in the world, a thorough curriculum on the way of operation should be done by concerned traffic departments of the cities through various means of traffic education. Better yet tough and strict penalties should be put in place for those found breaking traffic rules.

## 5. Conclusions

Saturation flow rate analysis was performed for a signalized intersection with the Special Width Approach Lane (SWAL) phenomenon. Previous models used the default saturation flow rate set by the Highway Capacity Manual which sometimes is not always accurate due to variance of influencing factors. Data were collected in SWAL based signalized intersections of Karlsruhe, Germany and an estimation model of the saturation flow rate is established. The comparison of the collected saturation flow rate and the calculated one shows the accuracy of the proposed model is about 95%. There is no much significant difference between them. According to the analysis, the following conclusions can be drawn.

1. The saturation flow rate model after performing several iterations converges at 1652 pcu/h/ln as the optimum saturation flow rate of the SWAL signalized intersection using the field data of the intersections in Karlsruhe, Germany.
2. The existence of cyclists and rainfall will further lead to a decrease in the saturation flow rate. The values of the adjusted parameters are 0.943 and 0.970, respectively.
3. Lane marking as an influencing factor is shown to improve the saturation flow rate. The value of the adjusted parameter are 0.986. However, the absence of lane marking in experienced drivers who use the road quite often is the same as being on a marked lane.

The survey shows that the SWAL works well in Karlsruhe, Germany. In each narrow lane, the saturation flow rate can be 1652 pcu/h/ln. However, it is not clear how much this has to do with the rigour of the Germans. If such design can be tested in other countries, we plan to make a comparison study, which can be helpful in improving the design of SWAL.
